# Pain and Psychological Readiness to Return to Sport in Elite Volleyball Players: A Cross-Sectional Study

**DOI:** 10.3390/ijerph20032492

**Published:** 2023-01-30

**Authors:** Rubén Gajardo-Burgos, Camila Valdebenito-Tejos, Germán Gálvez-García, Claudio Bascour-Sandoval

**Affiliations:** 1 Instituto de Aparato Locomotor y Rehabilitación, Facultad de Medicina, Universidad Austral de Chile, Valdivia 5090000, Chile; 2 Programa de Magister en Terapia Física, Mención Musculoesquelética, Universidad de La Frontera, Avenida Francisco Salazar 01145, Temuco 4780000, Chile; 3 Dirección de Desarrollo Estudiantil, Universidad de La Frontera, Avenida Francisco Salazar 01145, Temuco 4780000, Chile; 4 Departamento de Psicología, Universidad de La Frontera, Avenida Francisco Salazar 01145, Temuco 4780000, Chile; 5 Departamento de Psicología Básica, Psicobiología y Metodología de las Ciencias del Comportamiento, Facultad de Psicología, Campus Ciudad Jardín, Universidad de Salamanca, 37005 Salamanca, Spain; 6 Departamento de Ciencias de la Rehabilitación, Universidad de La Frontera, Avenida Francisco Salazar 01145, Temuco 4780000, Chile

**Keywords:** pain, psychological process, psychological readiness, volleyball, return to sport, athletic injuries

## Abstract

Pain is modulated by multiple factors. A relevant psychological process peculiar to athletes and which could be associated with pain is Psychological Readiness to Return to Sport (PRRS). The analysis of this association in competition context is particularly important. Objective: To determine the correlation between the PRRS and pain intensity in elite volleyball players during their participation in a continental sporting event. Methods: A cross-sectional study was conducted. Data from 107 male volleyball players (23.50 ± 4.08 years of age) participating in the South American Volleyball Championship were used. The athletes answered a self-report questionnaire on the day the championship began regarding their history of injuries in the previous six months. The athletes who declared injuries were asked about the current pain intensity using the Pain Numeric Rating Scale (NRS) and Psychological Readiness to Return to Sport using the Injury-Psychological Readiness to Return to Sport scale (I-PRRS). Results: 43.93% (*n* = 47) of the athletes (23.70 ± 3.54 years) reported an injury in the six months prior to the championship. They presented a median on the NRS of three (interquartile range (IQR), 2–5), and 54 (IQR, 46–58) on the I-PRRS. The Spearman’s Rho correlation test showed an inversely and moderate correlation (r_s_ = −0.36; *p* = 0.011; CI: −0.64–−0.08) between pain intensity and PRRS. Conclusions: In male elite volleyball players who participate in a Continental Championship in South America, higher levels of PRRS was correlated to lower pain intensity.

## 1. Introduction

Volleyball is one of the most popular sports in the world. At the competitive level it is physically demanding, causing sport injuries at different points in the athletes’ lives. In this sense, it has been demonstrated that volleyball-related injuries have an estimated incidence range from 1.7 to 10.7 injuries per 1000 playing hours, occurring more often during matches and among male players [1,2]. These injuries have physical and psychological repercussions for the athletes that can affect their participation in competitions and training. Many of the repercussions brought about by the injuries can present in the periods before a competition [3,4], possibly affecting the athlete’s performance in the competition.

One of the effects of sport injuries is pain. The current International Association for the Study of Pain (IASP) defines pain as an “unpleasant sensory and emotional experience associated with or resembling that associated with, actual or potential tissue damage” [5]. This protection mechanism [6] is modulated strongly by sensory, social, emotional, and cognitive factors [7,8], making its perception vary between different individuals and populations. A relevant factor to understand this difference between athletes and non-athletes is the former’s common coexistence with pain during training or competitions [9,10,11,12,13]. Thus, athletes may potentially perceive pain as a normal part of their preparation (i.e., no pain, no gain) and activity, and not necessarily as threatening information always related to an injury [14]. This may help explain that athletes who have suffered an injury return to sporting activity and even competition although they perceive pain [10]. However, the study of other psychological processes that could be associated with the perception of pain when returning to sport is still in the beginning stages. A relevant psychological process unique to athletes and which could be associated with pain is psychological readiness to return to sport (PRRS).

To date, various consensuses have shown the importance of PRRS [15,16,17]. This is de-fined as a psychosocial process which athletes may experience before, during or after their transition from rehabilitation to returning to competitive sport [18]. In the event of an injury, the athlete can respond with low psychological readiness, which can last throughout the rehabilitation process [19] and even once the athlete has returned to the sport [20]. Low PRRS is characterized, on the one hand, by reduced confidence, motivation, functional attention and unrealistic expectations—and on the other hand, by high levels of fear, anxiety or distrust in using the injured part [21]. In this sense, it must be emphasized that high levels of fear and anxiety have been related to greater pain intensity, both in healthy subjects and in subjects affected by pathologies or injuries [22]. Thus, given that fear and anxiety are important components of the PRRS, a low level of this could be associated with a pain perception of greater intensity.

In addition, elite athletes should be considered to participate in high-stress environment sports competitions. These can put the athlete in positive or negative psychological conditions, such as motivation or stress [23] which influences the PRRS, and therefore modifies their relation to pain. Thus, the PRRS takes on special relevance during competitions, since these are important milestones for elite athletes. In this vein, it has been reported that PRRS and pain affect performance during competitions [24]. However, the association between the two variables in the competitive period has not been explored.

Thus, the present study seeks to broaden knowledge of the correlation of psychological processes, particularly PRRS, and pain. Consequently, this study seeks to determine the correlation between PRRS and pain intensity in elite volleyball players during a competition. The first step is to assess this psychological process with respect to the perception of pain, contributing to how it is managed. This makes it possible to develop or strengthen strategies that seek a successful return to sport after having suffered an injury, highlighting in this process the importance of a holistic view.

## 2. Materials and Methods

The reporting of the paper follows the STROBE guidelines [25]. This is to ensure effective and clear communication of all the important aspects of this research.

### 2.1. Study Design

An observational, cross-sectional and analytical study was conducted on volleyball players on the national team. This study was approved by the Science Ethics Committee of the Valdivia Health Service.

The target population was male players participating in the XXXIII South American Volleyball Championship held in Temuco and Santiago in September 2019 in Chile. This study was approved and supported by the South American Confederation of Volleyball and the Volleyball Federation of Chile.

The aim and methodology of the study were presented to the coaches and delegates during the qualifying process of the teams in the two host cities. Next, a meeting was held with the athletes of the teams to inform them about the study and ask for their participation. If the athletes agreed to participate, they signed the informed consent and the evaluation instruments were applied. The evaluation instruments were applied by specially trained personnel.

All the players were invited to participate, i.e., the 110 athletes from the eight national teams of South America: Argentina (*n* = 14), Bolivia (*n* = 12), Brazil (*n* = 14), Chile (*n* = 14), Colombia (*n* = 14), Ecuador (*n* = 14), Perú (*n* = 14) and Venezuela (*n* = 14). The inclusion criteria were the following: be a registered athlete in the championship, have medical release to compete and have signed the informed consent. There were no exclusion criteria.

### 2.2. Procedure and Instruments

On the first day of the Championship, the athletes completed a hard copy self-report questionnaire with sociobiodemographic and sporting information such as age (years), body mass (kg), height (m), origin (country), playing position (libero, setter, outside spiker, spiker, and middle blocker), dominant hand (left/right/ambidextrous) and dominant leg (left/right/ambidextrous). The dominant hand was defined as the hand that is used for spiking or serving. The dominant foot was defined as the foot that is used to kick a ball. In this questionnaire, they also answered questions about the presence of injuries in the 6 months prior to the championship. For this, injury is defined as: “All musculo-skeletal injuries (traumatic and overuse) newly incurred during competition or training regardless of the consequences with respect to the athlete’s absence from competition or training” [26]. If the athletes did not report previous injuries the questionnaire was ended and they did not need to complete another one. However, if they reported an injury, they had to answer questions about the injury such as: duration (less than one month, between one and three months, three and six months), anatomical area and form of presentation of the injury (suddenly while performing normal training or competition or gradual onset, over several consecutive training sessions). Finally, they were asked about the current intensity of the pain with respect to their previous injury using the Numerical Rating Scale (NRS) [27] and the PRRS for sport today, through the self-report questionnaire Injury-Psychological Readiness to Return to Sport Scale (I-PRRS) [28,29].

The I-PRRS, developed by Glazer, is a self-administered 6-item questionnaire, considered easy to use, reliable and valid, to evaluate the PRRS of athletes to return to practice and competition participation after a sport injury and to measure the athlete’s confidence at a particular point in time [28]. The 6-items are: 1. My overall confidence to play is; 2. My confidence to play without pain is; 3. My confidence to give 100% effort is; 4. My confidence to not concentrate on the injury is; 5. My confidence in the injured body part to handle to demands of the situation is; and 6. My confidence in my skill level/ability is. Each of the items is answered on a scale of 100 points with an interval of one point. A score of 0 indicates that the athlete has no confidence and a score of 100 indicates that the athlete has complete confidence in the item asked about. To calculate the total score, the score of each of the six items was added and divided by 10. A consensus of a panel of experts has argued that this instrument is of great help to professionals in determining the level of psychological readiness of athletes to return to sport [15].

### 2.3. Statistical Analysis

The statistical analysis was performed with the Stata program v. 14. The continuous variables were reported according to measures of central tendency and dispersion. The categorical variables are presented in relative and absolute frequencies. The fulfillment of the assumption of normality of the continuous variables was evaluated using the Shapiro–Wilk test. Where the assumption of normality was not fulfilled (*p* < 0.05), the medians and interquartile range (IQR) were reported, and the Spearman’s rho coefficient (r_s_) was calculated to explore correlations between I-PRRS total (and each of its items) vs. pain intensity. The magnitude of the correlation was considered weak for 0.1, moderate for 0.3 and strong for 0.5 [30]. The analysis considered a statistical significance of 0.05.

## 3. Results

**Figure 1:**
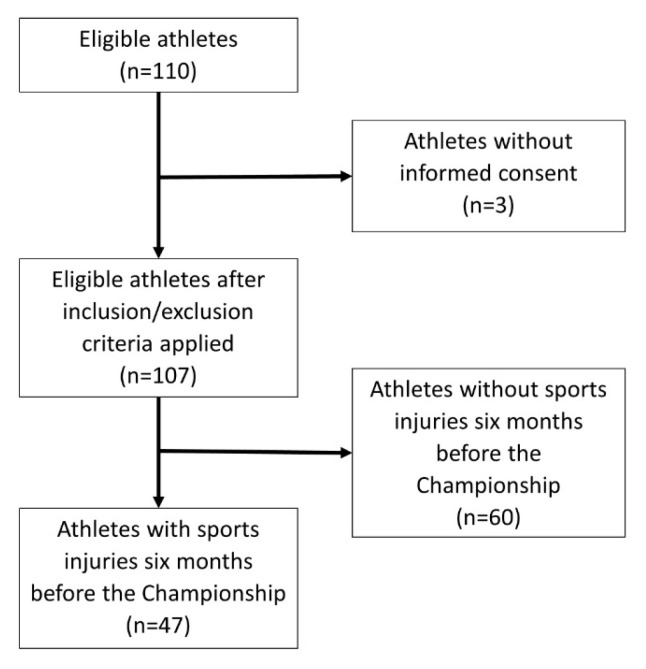
Flow diagram of athletes’ inclusion.

Of the 110 athletes, 97.3% (*n* = 107) agreed to participate and complete the questionnaires. Athletes of all the nationalities, i.e., the eight national teams, participated (23.50 ± 4.08 years old, 192.43 ± 9.00 cm of height, 87.30 ± 10.06 kg of weight) (see Figure 1).

Of all the participants, 47 players (43.93%) declared having had an injury in the six months prior to the Championship and having medical release to return to competition. All these players (*n* = 47) completed injury questionnaire, NRS and I-PPRS. Of these, 14 players (29.79%) suffered an injury in the month prior (NRS median 6, IQR 3–6), 20 (42.55%) between one and three months before (NRS median 3, IQR 2–3), and 13 (27.66%) between three and six months before the start of the Championship (NRS median 4, IQR 3–4). In the previously injured athletes, the NRS median was 3 (IQR, 2–5). The characteristics of the previously injured athletes are summarized in Table 1.

With respect to the anatomical location of the injury, 26 athletes reported an injury in the lower limb (55.32%), 14 in the upper limb (29.79%), five in the lumbar or abdominal area (10.63%) and two in other areas (4.26%). In particular, the most affected zones were the knee (*n* = 18, 38.30%) followed by the shoulder (*n* = 7, 14.89%). With respect to the onset of the injury, 26 reported suddenly while performing normal training or competition (55.32%) and 21 gradual onset over several consecutive training sessions (44.68%).

The results of the Injury-Psychological Readiness to Return to Sport scale (I-PRRS) are described in Table 2.

Items 3 and 5, and the total score on the I-PRRS are correlated negatively and significantly with the intensity of the pain (*p* < 0.05). See details in Table 3.

## 4. Discussion

The aim of this study was to determine the correlation between PRRS and pain intensity in elite male volleyball players during a competition. Our results showed that higher levels of PRRS was correlated to lower pain intensity in this group of athletes. This is relevant given that both factors may affect performance during a competition [24]. To the best of our knowledge, this is the first study to analyze this correlation in athletes during a competition.

We demonstrate an inverse and moderate correlation between pain intensity and PRRS. This result is in line with a recent study conducted by Sala-Barat et al. [31] on individuals subjected to an anterior cruciate ligament reconstruction. They found that higher levels of psychological adaptation for the return to sports practice was related to lower pain intensity, with the magnitude of this correlation being moderate (i.e., r_s_ = 0.4) as in our study. In addition, it is worth noting that the scale used in the study by Sala-Barat et al. [31] (i.e., Spanish version of the anterior cruciate ligament-return to sport after injury) showed a strong correlation (i.e., r = 0.8) with the I-PRRS used in our study. The inverse correlation between pain intensity and the I-PRRS could be explained in three ways. First, during the injury period, particularly in the final recovery process, the athlete is commonly exposed to significant external pressure (i.e., trainers, team mates, family and mass media) as well as internal pressure (i.e., athletic identity, compensation, guilt) [32] which may lead to high levels of emotional anxiety and catastrophic thinking. These have been linked to the presence of greater pain intensity [33], which could be interpreted by the athlete as the presence of an injury despite having medical release, developing into a reduction in their PRRS. Consequently, elevated pain intensity could result in lower confidence while competing. Second, a high PRRS could be related to high self-efficacy for managing pain, which would involve a lower perception of pain [22]. Third, in the context of an important sporting competition there is high motivation to participate in the event, especially when they are very important. This carries with it a positive perception of returning to the sport [16], leading to high PRRS. Thus, the athlete would perceive the return to the sport as less threatening and may consequently have a lower perception of pain. However, these proposed reasons must be confirmed in future studies.

The correlation between pain intensity and the items on the scale highlights that high pain intensity is associated with low confidence in giving 100% of their effort. This could be related to competition being perceived as uncontrollable and unpredictable, forcing the athlete to give 100% effort at any time [34], and the presence of high pain intensity would prevent the athlete from performing certain movements, limiting the range of these movements and undermining the athlete’s confidence in his ability.

The association between pain and confidence in the injured area may be explained by the nature of competitive sport. Volleyball demands a large repertoire of movements [35] which is why one movement of a joint in particular could be compensated by other joints. Thus, it is possible to return to competition despite not trusting the segment in particular. However, during the competition, the need to make a greater effort and achieve limited movements could generate a perception of threat when moving the injured segment and this increases the perception of pain. In the same vein, it must be noted that pain from an injury is perceived in a limited area of the body and if its perception is interpreted as an alarm, it can reduce confidence in the use of the segment. It must be emphasized that during the creation of the instrument, this item was assessed positively by experts since according to them, this item is the most relevant for PRRS [28].

A remarkable finding was the presence of pain one day before the competition in 41 (38.32%) athletes, despite having medical release. One condition that recurs in this population—as it is often one of the measures to achieve their sporting, economic and social objectives—is to compete in spite of the pain [9]. In our case, the athletes were facing a Continental Championship that takes place every two years and that allows them to face the best teams on the continent. In addition, this championship offered the opportunity to qualify for the Olympic Games, so it was expected that the athletes would be highly motivated. These results could indicate that the athletes, in spite of their motivation, could think they can participate in the game to a large extent without pain or with pain that allows them to perform their sport, but they do not trust they can give 100% effort and demand from the injured body part as a result of the pain, a highly likely situation during a competition of this type.

Several studies have reported that the physical variables do not account for the athlete’s overall condition to return to the sport [36,37]. What is more, non-optimal psychological states [38] reduce the possibility of a successful return and increase the risk of a new injury [16]. This is especially relevant in the context of sporting competitions, where the athlete must also cope with the physical demand imposed by the competition as well as the psychological demand. In this vein, it has been reported that PRRS and pain affect performance during competitions [24,39]. This underscores the importance of the periodic assessment and management of psychological processes, with special emphasis prior to competitions, by the health and coaching teams. In the same way, the correct management of the pain by the health team could improve the athlete’s confidence, and promoting the PRRS could have a positive impact on the perception of pain. Thus, for instance, pharmacological treatment should only be considered one of the components of pain management and treatment [40]. However, a comprehensive approach should be considered, including physical aspects (i.e., individualized exercise plan, biomechanical factors, load progression, etc.) and psychological (i.e., sleep quality [41], coping strategies, self-efficacy, motor imagery, and confidence). It has been identified that confidence is one of the most important components of the PRRS [42], being an essential element in sporting success [15,17]. For example, developing the athlete’s confidence in the injured part of the body, particularly with the use of target-setting strategies with respect the return to the sport (i.e., realistic goals, while also encouraging the player to accept they may still have limitations), could aid in a good transition to the return to competition [43]. This strategy is based on (1) evaluating the athlete globally (beliefs, objectives and attitudes), (2) setting a realistic and meaningful goal for the athlete, and (3) establishing clear specific objectives that allow the development of the general objective [44,45] (for more details, see Roberts & Kristiansen). This way, the evaluation of interventions that seek to improve PRRS, such as behavioral cognitive therapy or motivational interviews, could be relevant to improving the effectiveness of the treatment of painful syndromes or dysfunctions. Thus, future studies should include longitudinal designs and cognitive variables (i.e., catastrophism, self-efficacy) and emotional variables (i.e., distresses) and analyze their association with PRRS.

This study has some limitations. The cross-sectional nature of the study prevents conclusions from being drawn as to the causal directionality of our findings, making more studies necessary to clarify this association. Moreover, no calculation was made of the sample size due to the complexity of gaining access to these athletes during a Championship. In spite of this, we emphasize that 97.3% of the athletes registered agreed to participate in this study. The severity of the injury can also be a confounding factor when examining the return to the sport. The severity of the injury or pain treatments can also be a confounding factor when examining the return to the sport. Regarding the first, the athletes with more serious injuries can show more prolonged negative psychological responses of greater magnitude [16]. This information could not be compiled given the limited access time to the athletes. The sample was comprised of a homogenous group of athletes—elite Latin American male volleyball players—which means they have particular characteristics. Therefore, extrapolation of these results to other populations must be done with caution. However, considering the lack of studies in the field, we believe this study could serve as a basis for future studies. Furthermore, these future studies should include larger sample sizes along with probability sample designs, which would make it possible to generalize the findings and consider variables that could modify the association between pain and psychological readiness, such as severity of injury, type of treatment, etc.

## 5. Conclusions

In male elite volleyball players who participate in a Continental Championship in South America, there is an inverse and moderate correlation between the PRRS and pain intensity, that is, higher levels of PRRS was correlated to lower pain intensity. Our results show an aspect to be taken into account in the management of athletes with pain who return to competition. These findings must be considered by the health and coaching teams since both variables are potentially modifiable and may impact on the athletes’ performance during a competition. Furthermore, we believe that new studies are needed to understand the effect of the intervention in PRRS on pain, and vice versa, in elite athletes, as well as studies that make it possible to understand the role of PRRS in managing pain in elite athletes.

## Figures and Tables

**Table 1:** Physical and sport characteristics of the athletes with previous injury.

	M ± SD	*n* (%)
Age (years)	23.70 ± 3.54	
Height (cm)	192.11 ± 8.48	
Body mass (kg)	87.70 ± 9.83	
BMI	23.76 ± 2.18	
Game position		
Setter		7 (14.89)
Middle blocker		13 (27.66)
Outside spiker		9 (19.15)
Spiker		14 (29.79)
Libero		4 (8.51)
Dominant hand		
Right		44 (93.62)
Left		3 (6.38)
Dominant leg		
Right		39 (82.98)
Left		8 (17.02)

M = mean, SD = Standard deviation, *n* = number of participants, % = percentage, BMI = Body mass index.

**Table 2:** Injury-Psychological Readiness to Return to Sport Scale.

Item	Md (IQR)
1. My overall confidence to play is	100 (90–100)
2. My confidence to play without pain is	90 (60–100)
3. My confidence to give 100% effort is	100 (99–100)
4. My confidence to not concentrate on the injury is	90 (50–100)
5. My confidence in the injured body part to handle to demands of the situation is	85 (60–100)
6. My confidence in my skill level/ability is	100 (80–100)
I-PRRS total	54 (46–58)

Md = Median, IQR = Interquartile range.

**Table 3:** Correlation of the Injury-Psychological Readiness to Return to Sport Scale with Pain Numerical Rating Scales.

Items	r_s_ (95% CI)	*p*-Value
1. My overall confidence to play is	−0.02 (−0.33–0.29)	0.898
2. My confidence to play without pain is	−0.02 (−0.50–0.08)	0.162
3. My confidence to give 100% effort is	−0.37 (−0.061–−0.12)	0.003 *
4. My confidence to not concentrate on the injury is	−0.24 (−0.53–0.04)	0.093
5. My confidence in the injured body part to handle to demands of the situation is	−0.54 (−0.78–−0.29)	<0.001 **
6. My confidence in my skill level/ability is	0.01 (−0.31–0.34)	0.947
I-PRRS total	−0.36 (−0.64–−0.08)	0.011 *

r_s_ = Spearman’s rho, 95% CI = 95% Confidence interval; * significant moderate correlation; ** significant strong correlation.

## Data Availability

The data that support the findings of this study are available from the corresponding author, upon reasonable request.
